# Joint Source and Channel Rate Allocation over Noisy Channels in a Vehicle Tracking Multimedia Internet of Things System

**DOI:** 10.3390/s18092858

**Published:** 2018-08-30

**Authors:** Yixin Mei, Fan Li, Lijun He, Liejun Wang

**Affiliations:** 1 School of Electronic and Information Engineering, Xi’an Jiaotong University, Xi’an 710049, China; xamichelle@stu.xjtu.edu.cn (Y.M.); jzb2016125@mail.xjtu.edu.cn (L.H.); 2 College of Software, Xinjiang University, Urumchi 830046, China; wljxju@xju.edu.cn

**Keywords:** multimedia IoT, video analysis, vehicle tracking, rate allocation, surveillance system, forward error correction

## Abstract

As an emerging type of Internet of Things (IoT), multimedia IoT (MIoT) has been widely used in the domains of healthcare, smart buildings/homes, transportation and surveillance. In the mobile surveillance system for vehicle tracking, multiple mobile camera nodes capture and upload videos to a cloud server to track the target. Due to the random distribution and mobility of camera nodes, wireless networks are chosen for video transmission. However, the tracking precision can be decreased because of degradation of video quality caused by limited wireless transmission resources and transmission errors. In this paper, we propose a joint source and channel rate allocation scheme to optimize the performance of vehicle tracking in cloud servers. The proposed scheme considers the video content features that impact tracking precision for optimal rate allocation. To improve the reliability of data transmission and the real-time video communication, forward error correction is adopted in the application layer. Extensive experiments are conducted on videos from the Object Tracking Benchmark using the H.264/AVC standard and a kernelized correlation filter tracking scheme. The results show that the proposed scheme can allocate rates efficiently and provide high quality tracking service under the total transmission rate constraints.

## 1. Introduction

The Internet of Things (IoT) is a global infrastructure for the information society, enabling the advanced services by interconnecting physical and virtual things based on the existing and evolving interoperable information and communication technologies [1]. The IoT extends the conventional concept of the Internet as an infrastructure network reaching out to end-user’s terminals, to a concept of a pervasive network of interconnected objects. As an emerging type of IoT, Multimedia IoT (MIoT) refers to IoT with multimedia outputs or/and inputs [2]. MIoT has been widely used in the domains of healthcare [3,4], smart buildings/homes [5,6], transportation [7,8] and surveillance [9], etc. Particularly, intelligent surveillance networks are some of the main applications of MIoT. They normally consist of sensors, a transmission network and server [2]. The sensors and the server carry out the tasks of capturing and processing the videos, and the transmission network is responsible for the transmission of all kinds of information. Although a great proportion of videos are captured for human consumption, more and more videos are transmitted for video analysis rather than being processed manually in intelligent surveillance networks. In a realistic surveillance MIoT with the purpose of video analysis, videos are uploaded to a server to meet the computation capability requirement of video processing and analysis. When the video sequences are uploaded via a wireless network, multiple camera nodes compete for the limited transmission resources. Due to the huge amount of video data and the limited wireless resources, it is challenge to allocate the limited resources efficiently based on the video content features to achieve high quality intelligent surveillance service for MIoT systems. 

A lot of existing research has considered video coding and transmission schemes to improve the human visual quality [10,11,12,13,14,15]. The authors in [10] proposed a joint coding and transmission scheme to provide high quality video for human consumption. First, the video coding and transmission scheme was modified to get higher structural similarity index (SSIM). Then in a server, the multiple videos were merged to one video with a wide viewing angle and high resolution, to provide high quality surveillance video for human consumption. In [11], a background-model-based wireless video transmission scheme was proposed to provide high quality of experience (QoE) for users. The foreground (like pedestrians) regions were detected in camera mode. Then they were uploaded to server instead of full video sequences to avoid the degradation of QoE caused by limited transmission resources. Considering the channel fading and shadowing, [13,14,15] employed Gilbert-Elliot model to estimate the burst-loss prone channel condition and avoid the corrupt decoding caused when bursts of noises occur. All these schemes were designed to improve the quality of service/experience to provide better viewing experiences for clients. However, none of them are suitable for a surveillance network with the purpose of video analysis. 

To improve the service quality of video analytics in surveillance networks, plenty of researchers have worked on improving the performance of video analysis schemes in the computer vision field. Detection, recognition and tracking of human/vehicles are the main analysis tasks of smart surveillance systems [16,17,18,19,20]. In [21], an object detection algorithm was proposed based on the discriminatively trained part models. In [22], an object detection scheme was proposed based on region proposal networks. Minimum Output Sum of Squared Error filter (MOSSE) is a correlation filter-based tracker, which firstly uses a correlation filter for object comparison [23]. In [24], a kernel trick was used to improve correlation filters, which turned the linear classifier into a non-linear ridge regression classifier. Based on [24], Henriques et al. proposed a kernelized correlation filter (KCF) tracking scheme, which employed histogram of oriented gradient (HOG) features rather than gray scale features [25]. These works are representative video analysis schemes, which possess in common the characteristic of high computation complexity. Therefore, it is better to assign these complex video analysis tasks to a server, which has greater computational capacity than camera nodes.

The main challenges for the schemes which chose to upload videos and analyze videos on a server, are the limited transmission resources and network state fluctuations. In [26,27], video features rather than full video sequences were uploaded to the server for analysis to save transmission resources. However, the full videos are not accessible on the server, which limits the further investigation. In [28], full video sequences were uploaded to a server for analysis. However, the video analysis performance degradation caused by compression or transmission errors are not considered.

Only a few researchers have studied the effect of video compression and transmission parameters on video analysis performance. In [29], a saliency-based rate control scheme was proposed for human detection with a single camera. A standard-compliant video encoding scheme was proposed to obtain better object detection performance on compressed video [30]. In [31,32], the effect of quantization parameter (QP) and transmission conditions on human detection were considered to optimize the performance of human detection in server. However, due to the lack of the consideration of video content, which is closely related to the analysis performance, the prediction of human detection precision is not accurate.

In a realistic surveillance network for vehicle tracking, all surveillance videos are uploaded to server for video analysis purposes. When video sequences are uploaded via a wireless network, multiple camera nodes compete for the transmission resources which are limited by the bandwidth. Therefore, considering the effect of video content and video quality degradation caused by compression and transmission error, a joint source and channel rate allocation scheme is proposed in this paper to optimize performance of KCF tracking scheme in server. The main contributions of this paper can be summarized as follows:

(1) Content-aware tracking precision prediction model. The features of the video content, which are not considered in existing prediction models, have a great impact on tracking precision. Based on the effect of video compression and video content on KCF tracking scheme, we take the bits per pixel, luminance values and complexity of the video content as key factors in the process of HOG extraction and object matching in KCF scheme. Then, based on the obtained relationship between these factors and the tracking precision, we establish a content-aware tracking precision prediction model by using curve-fitting method. 

(2) Tracking-precision-driven joint source and channel rate allocation scheme. Due to the limited wireless resources and the unreliability of wireless networks, it is important to design a joint source and channel rate allocation scheme to avoid degradation of tracking precision caused by video compression or transmission errors. We optimize the rate allocation for camera sensors by a tracking-precision-driven optimization problem. Based on our content-aware tracking precision prediction model, the optimization problem is formulated to maximize the product of tracking precision of each surveillance video in MIoT server under the constraint of the total transmission rate in the wireless network. To improve the reliability of data transmission and the real-time of the video communication, we adopt forward error correction (FEC) in the application layer. 

The rest of this paper is organized as follows: Section 2 introduces the scenarios and structure of our MIoT system for vehicle tracking. Section 3 presents a content-aware tracking precision prediction model. The proposed joint source and channel rate allocation scheme are given in Section 4. The simulation results are shown in Section 5, followed by the conclusions in Section 6.

## 2. Scenarios and Structure of MIoT System for Vehicle Tracking

**Figure 1:**
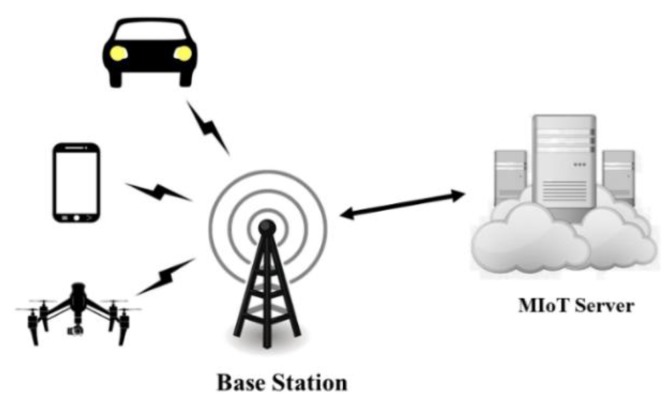
Scenario of the MIoT system.

Figure 1 shows the scenario of the MIoT system. In the MIoT system, multiple mobile camera nodes are distributed and move round randomly. A mobile camera node can be a mobile sensor device like a car recorder, smart phone or unmanned aerial vehicle, etc. Every mobile camera node can capture, encode, and upload videos to the MIoT server via a wireless network. The MIoT sever analyzes the received videos with a tracking algorithm once the tracking tasks are assigned. Due to the mobility and random distribution of camera nodes, wireless wide area networks (WWANs) are employed for video transmission. In WWAN, mobile nodes in a large geographic area can establish radio frequency links with the base station, and the base station establishes global connectivity to the backbone core network [33]. 

**Figure 2:**
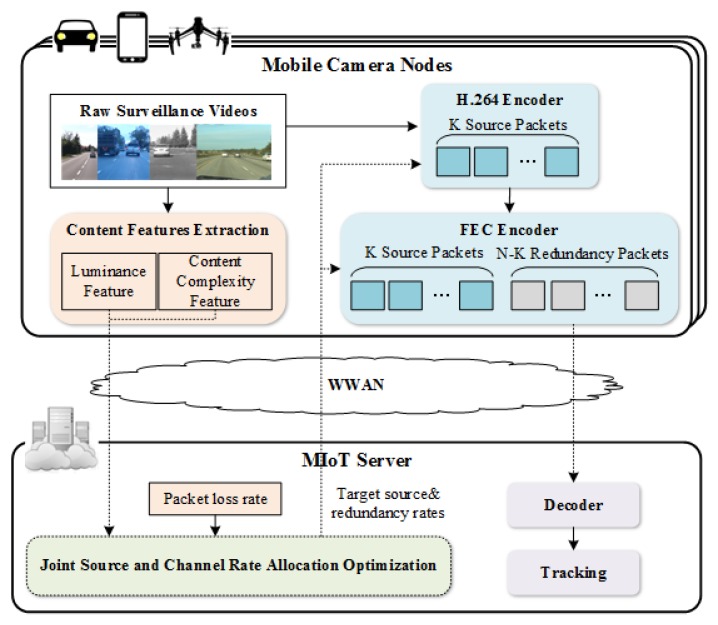
Structure of MIoT system for vehicle tracking.

The proposed structure of the MIoT system for vehicle tracking, which consists of three main components, namely a mobile camera node, wireless network and MIoT server, is shown in Figure 2.

Mobile camera node: This can be a mobile sensor device like a car recorder, smart phone, or an unmanned aerial vehicle, etc. In every group of picture (GOP) period, the camera node has two tasks: (1) The camera node calculates and uploads video content features of current uncoded GOP to a rate allocation module in the server; (2) the camera node encodes and uploads the GOP according to the rate allocation policy from the MIoT server. The H.264 encoder and Raptor [34] FEC encoder are applied for video coding in each camera node.

Wireless Network: A wireless wide area network (WWAN) is chosen for video transmission due to the random distribution and mobility of camera nodes. When uploading videos to the server via a WWAN, multiple camera nodes compete for transmission resources which are limited by the bandwidth. Due to the inherent transmission characteristics of wireless networks, packet loss occurs in the transmission network. In this paper, we assume that the packet loss rate of each camera node is perfectly known by the MIoT server.

MIoT server: In the MIoT server, a joint source and channel rate allocation module optimizes the source and FEC rates for each camera node according to the content features and the current channel packet loss rates of GOPs in all camera nodes. Then the target source and redundancy rates of all camera nodes are sent back to each camera node for current GOP encoding. Besides, the MIoP server stores or performs vehicle tracking on received videos according to the actual demand. Since the tracking tasks usually are assigned with a delay in realistic scenarios, there is no real-time feedback about the tracking results in the MIoT server.

The FEC encoder shown in Figure 2 is an application layer FEC encoder. Application layer forward error correction solutions provide a straightforward and powerful means to overcome packet loss [35]. At the camera node, for every *K* source packets of video stream, (*N*−*K*) redundant data packets are produced by the FEC encoder and are sent with source packets of the source block. As long as the receiver, which is a MIoT server in our structure, receives at least any *K* of the *N* packets, it can recover all the source packets [36].

## 3. Content-Aware Tracking Precision Prediction Model

### 3.1. Factors Impact on KCF Tracking Scheme

The KCF tracking scheme is proposed by Henriques et al. in 2015 [21]. Since KCF is a well-accepted, robust, and computationally efficient scheme, it is adopted as the vehicle tracking scheme in the MIoT server.

**Figure 3:**
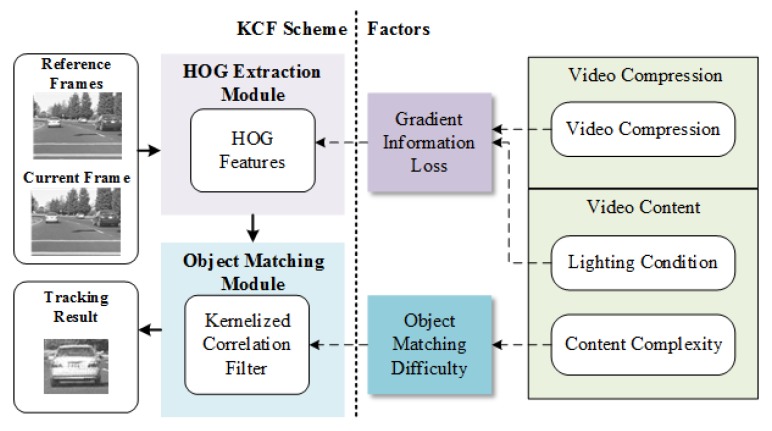
Factors impact on HOG extraction and object matching in KCF scheme.

As shown in Figure 3, The KCF tracking scheme includes two important processes, HOG extraction and object matching. In HOG extraction module, HOG features of reference frames and current frame are extracted to represent the shape of objects. The HOG features are calculated by orientation and magnitude of the intensity gradient of pixels, which can be modified by the compression rate and video lighting conditions. In the object matching module, the HOG features in the reference frames are trained to get the kernelized correlation filter. Then the HOG features of the current frame are correlated with the filter to determine the object position in current frame. The correlation responses are based on the shapes of target object and its surroundings in the frames, which are determined by the video content complexity. Therefore, HOG extraction and object matching are the two modules which are sensitive to video compression and video content.

### 3.2. Model Features Extraction and Analysis

#### 3.2.1. Bits per Pixel

Since HOG features can be affected by the artifacts created by video encoder at different compression rates [29], we employ the bits per pixel to represent the effect of video compression on HOG computation. Videos with different resolution require different coding rates to provide high video quality. Due to the variety of resolution of mobile camera devices, bits per pixel instead of bit rate is employed to maintain the quality of different resolution videos. Bits per pixel (*B_pp_*) can be calculated as follows:

(1) $$B_{pp}=\frac{r}{f_{ps}\cdot N_{px}},$$

where *r*, $f_{ps}$ and $N_{px}$ are the coding bit rate, frame rate and the number of pixels in a frame, respectively.

#### 3.2.2. Video Luminance Level

When a camera is capturing video, the luminance value of pixels can be greatly affected by the lighting conditions at that time. Under the circumstance of weak lighting conditions, cameras are prone to lose detailed information of objects because of the lack of reflected light from objects and the interference of noise, and HOG features are affected consequently. We employ luminance level (*L*) to represent the effect of lighting conditions on HOG computation.

**Figure 4:**
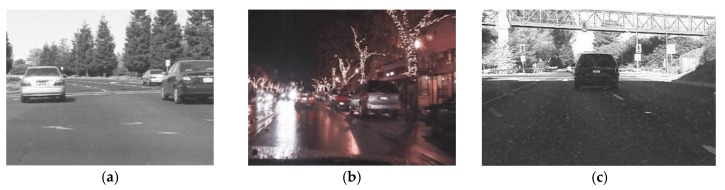
Examples of different video scenarios: (**a**) Open field in daytime; (**b**) Open field at night; (**c**) Covered field in daytime.

We consider two scenarios: the open field and the covered field. The open field frame is the frame which has scenes of a vehicle driving in an open field, such as Figure 4a,b. In Figure 4a, vehicles are driving in an open field with strong lighting conditions during daytime, and all frames have similar and high luminance levels. In Figure 4b, vehicles are driving at night, all frames in this video sequence maintain a relatively stable low luminance level. In the open field frame, most parts of the frame have similar luminance levels. The covered field frame is the frame which has scenes of a vehicle driving in the field covered with a roof or canopy in daytime, such as Figure 4c. In Figure 4c, although the videos are captured during daytime, the luminance levels of the in tunnel regions and out of tunnel regions are different. Therefore, in a covered field frame, the largest region has a low luminance level because of the roof or canopy and the other uncovered field regions have high luminance levels.

**Figure 5:**
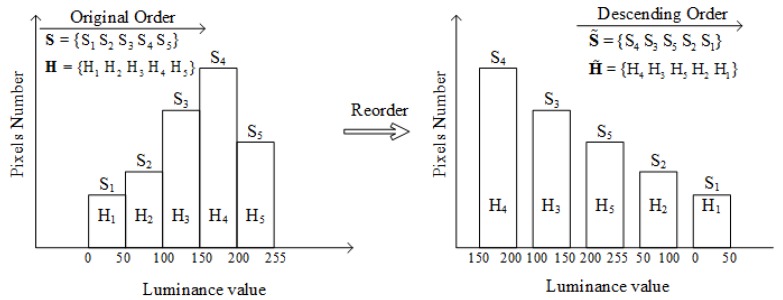
The histogram of pixels luminance values and the reordered histogram.

The open and covered field frames are classified by the statistical results of luminance of the pixels in a frame. The histogram of luminance values of pixels in a frame is obtained as shown in Figure 5. The luminance values are sorted into five intervals. The quantitative values of each interval are denoted as **H** = [25 75 125 175 225]. The numbers of pixels in all intervals are set as S. Then, as shown in Figure 5, the histogram is re-ordered by the descending order of the numbers of pixels $\tilde{S}$, and the corresponding quantitative values are set as $\tilde{H}$. To exclude the singular pixels of a frame, only first three elements of $\tilde{S}$ and $\tilde{H}$ are used in frame classification method and luminance level calculation method. The frame *f* can be classified based on reordered luminance histogram as follows:

(2) $$\left\{ \begin{array}{l} f\in C\;if\;{\tilde{H}}_{1}<T_{L1}\;and\;{\tilde{H}}_{2}>T_{L2}\\ f\in O\;otherwise \end{array}\right.,$$

where ***C*** and ***O*** indicate the set of covered and open field frames, respectively. $T_{L1}$ is the luminance threshold for dark regions and $T_{L2}$ is the luminance threshold for bright regions. The first two intervals [0,50] and (50,100] are considered as dark, and the last two intervals (150,200] and (200,255] are considered as bright. Hence, $T_{L1}$ and $T_{L2}$ are set to be 100 and 150, respectively. Although fixed thresholds $T_{L1}$ and $T_{L2}$ are employed to improve the operation speed, this method can be improved by employing dynamic thresholds instead of fixed thresholds. In different scenarios, the division of bright and dark regions can be different, so dynamic thresholds can better adapt to changes of scenarios and provide more accurate classification results of bright and dark regions. Hence, the interference regions can be excluded precisely.

Then the luminance level of frame can be obtained as follows:

(3) $$l(f)=\left\{ \begin{array}{l} \frac{\sum_{i=1}^{3}u\left({\tilde{S}}_{i}\right){\tilde{H}}_{i}{\tilde{S}}_{i}}{\sum_{i=1}^{3}u\left({\tilde{S}}_{i}\right){\tilde{S}}_{i}}\;if\;f\in C\\ \frac{\sum_{i=1}^{3}{\tilde{H}}_{i}{\tilde{S}}_{i}}{\sum_{i=1}^{3}{\tilde{S}}_{i}}\;if\;f\in O \end{array}\right.,$$

where:

(4) $$u(x)=\{\begin{array}{cc} 1 & if\;x\leq T_{L1.}\\ 0 & if\;x>T_{L1} \end{array}.$$

As in Equation (3), in the covered field frames ***C***, the bright regions are excluded for calculating the luminance level, since the vehicles are actually in the dark region with a low luminance level. In the open field ***O***, all regions represented by ${\tilde{H}}_{1}$, ${\tilde{H}}_{2}$ and ${\tilde{H}}_{3}$ are considered.

Based on the luminance levels of classified frames, the video scenarios can be detected and the video luminance level is obtained consequently. According to the previous description about scenarios, the video that has several consecutive covered field frames is regarded as a covered field scenario. Therefore, a video with more than five consecutive covered frames is considered a covered field video. The other videos are considered as open field videos. 

Due to the error propagation caused by the tracking scheme, different luminance level calculation methods are designed for different scenarios. In the tracking scheme, once a wrong target is determined in a frame due to the loss of HOG information caused by a low luminance level, the right target is hard to choose in all following frames even when the following frames have high luminance levels. Therefore, the open field frames are excluded when calculating the luminance level of video that has daytime covered field scenarios, as shown in Equation (5):

(5) $$L=\frac{1}{N_{c}}\sum_{f\in C}\left(\omega (f)\cdot l(f)\right).$$

The other videos, which have open field scenarios in daytime or at night have a different luminance level calculation method, that is:

(6) $$L=\frac{1}{N}\sum_{f\in C\cup O}\left(\omega (f)\cdot l(f)\right),$$

where l(f) is the luminance level of the *f*-th frame, *N* is the total frames in the video sequence. *N_c_* is the total covered field frames in the video sequence. Hence, ω(f) is the weight of the luminance level of *f*-th frame in video which can be obtained by Equation (7):

(7) $$\omega (f)=\frac{N-f+1}{N},$$

here ω(f) is determined by the number of frames influenced by the *f*-th frame. In other words, ω(f) is determined by the number of frames following the current frame.

#### 3.2.3. Video Adjacent Block Difference

In the KCF tracking scheme, the correlation results from kernelized correlation filter depend on the shapes of objects in the frame. In a video with complex content, the target object is surrounded by other background objects and the target object is hard to separate from the surroundings, which decreases the matching precision. We employ the video adjacent block difference (*V*) to represent the effect of video content complexity on tracking precision.

The adjacent block difference of a video sequence is defined as the maximum frame adjacent block difference just as follows:

(8) V=max(v(f)),

where v(f) represents the frame adjacent block difference of the *f*-th frame, defined as:

(9) $$v(f)=\frac{N_{clst}(f)}{N_{fmb}(f)},$$

where *N_fmb_*(*f*) is the total number of foreground MBs in the *f*-th frame, and *N_clst_*(*f*) is the total cluster number of the *f*-th frame, which can be obtained based on the cluster results of foreground macroblocks (MBs) in the *f*-th frame. Here, the foreground MBs refers to the MBs which have more edges than other MBs in the frame. As the analysis in Section 3.1 shows, the target is tracked based on the correlation results of the HOG features of the target and its surroundings. Complex HOG features have a higher probability to affect the tracking precision. Therefore, the MBs with relatively complex HOG are detected by gradients of pixels in the following. Since these complex HOG are almost caused by edges of objects, we call the corresponding MBs foreground MBs. The foreground MBs are detected based on magnitudes of the intensity gradients of all pixels in an image. The frame is divided into lots of non-overlapping 16 × 16 MBs. Let G(x,y) be the average magnitudes of intensity gradients of all pixels in MB(x,y). The *G_min_* is the minimum of G(x,y), and the *G_mean_* is the mean magnitudes of intensity gradients in a frame. Then foreground MBs are detected based on the gradients magnitudes in the frame. Objects, such as vehicles, trees and pedestrians, always have more edges than background like road and sky. Then, we define the foreground set Ω as:

(10) $$\left\{ \begin{array}{l} \mathrm{MB}(x,y)\in \Omega \;if\;G(x,y)>T_{m}\\ \mathrm{MB}(x,y)\notin \Omega \;otherwise \end{array},\right.$$

where:

(11) $$T_{m}=(G_{mean}+G_{\min})/2.$$

**Figure 6:**
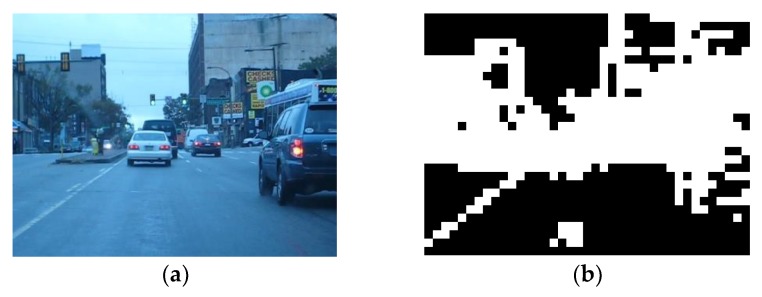
Sample of foreground detection result: (**a**) Sample frame; (**b**) Foreground detection result of sample frame.

Then, *N_fmb_* is defined as the total number of MBs in Ω. A sample frame and its foreground is shown in Figure 6, the white block is foreground MB and the black block is background MB.

**Figure 7:**
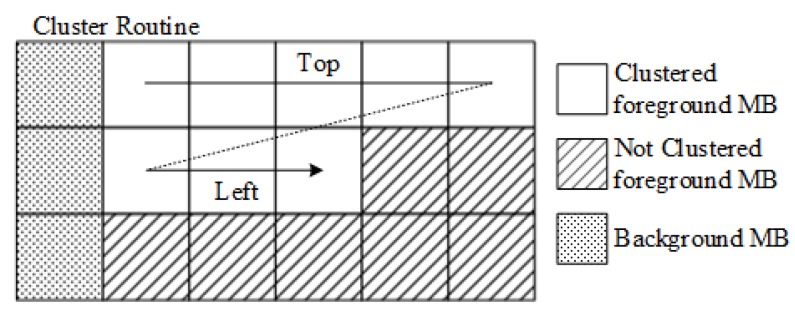
The cluster routine and available adjacent macroblocks.

The total cluster number *N_clst_* is then obtained based on clustering of foreground MBs. As shown in Figure 7, each foreground MB is compared with its clustered adjacent foreground MBs based on the average block luminance values. MBs that have similar average luminance level are clustered. Due to the clustering routine shown in Figure 7, only top or left foreground MB may be available for clustering the current MB. Let c(x,y) and I(x,y) be the cluster number and average luminance value of MB(x,y), respectively. The cluster number of the first foreground MB in frame is set to 1. Then the cluster number of foreground MB(x,y) is obtained as follows:

(12) $$c(x,y)=\left\{ \begin{array}{l} c(x-1,y)\;if\;\Delta_{1}<T_{\Delta }\;and\;\Delta_{1}\leq \Delta_{2}\\ c(x,y-1)\;if\;\Delta_{2}<T_{\Delta }\;and\;\Delta_{2}<\Delta_{1}\\ \max(c)+1\;otherwise\; \end{array},\right.$$

where max(*c*) is the maximum cluster number when the MB(x,y) is being clustered, and max(*c*) + 1 indicates that the MB(x,y) is considered as a new cluster. The parameter $T_{\Delta }$ is threshold of similar adjacent MBs, which is set as 20 in this paper. $\Delta_{1}$, $\Delta_{2}$ are defined as:

(13) $$\left\{ \begin{array}{l} \Delta_{1}=\left|I(x,y)-I(x-1,y)\right|\\ \Delta_{2}=\left|I(x,y)-I(x,y-1)\right| \end{array}\right..$$

Then, *N_clst_* is defined as the maximum *c* in the frame. In one cluster, magnitudes of gradients between adjacent MBs are prone to be small, which makes object matching difficult. Thus a smaller *N_clst_* indicates more complex content.

### 3.3. Model Establishment

**Figure 8:**
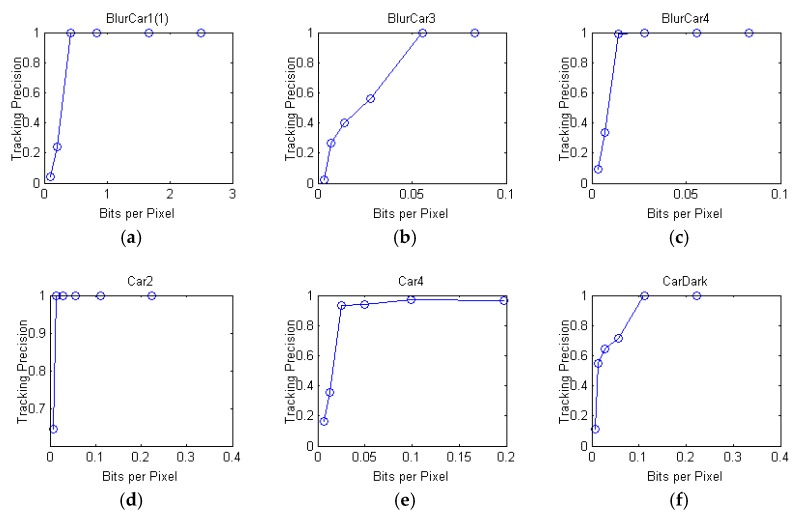
Tracking precision of each video sequences: (**a**) BlurCar1(1); (**b**) BlurCar3; (**c**) BlurCar4; (**d**) Car2; (**e**) Car4; (**f**) CarDark.

We select six videos which take vehicles as the target objects from the Online Object Tracking Benchmark (OOTB) [37]. Among them, BlurCar1 is divided into two video sequences. BlurCar(1) is a video sequence which is clipped from 1-st frame to 320-th frame in BlurCar1, and BlurCar(2) has the 390-th frame to 709-th frame in BlurCar1. Each video is coded at different bit rates using the H.264 coder as shown in Table 1. The target bit rate of each frame within the GOP is assigned using the basic variable bit rate mechanism in the H.264 coder. The frame rate is 30 fps, and the Group of Picture (GOP) structure and size are IPPP and 16, respectively. The tracking precision of each coded video sequence is obtained by KCF tracking scheme and is shown in Figure 8. Tracking results are compared with the ground truth. In each frame if the distance between center of tracking result and center of ground truth is less than 20, then the tracking result is considered as correct [25]. The tracking precision is the number of correct frames divided by number of all frames.

We use *B_pp_* to be our main tracking precision prediction parameter. According to the relationship between tracking precision and *B_pp_* shown in Figure 8, the tracking precision can be formulated as:

(14) $$P(B_{pp})=\frac{1}{1+\alpha e^{-\beta \cdot \sqrt{B_{pp}}}},$$

where *P* is the tracking precision, and α, β are empirical parameters. We can determine the shape of the prediction function according to α and β. As interpreted in Figure 8, the shape of the prediction function varies according to different video content. Therefore, α and β are estimated by *L* and *V* of the video as follows:

(15) $$\begin{array}{l} \alpha =a_{1}\cdot L^{a_{2}}\cdot V^{a_{3}}\\ \beta =\min\{a_{4}\cdot {\left(a_{5}\cdot L+a_{6}\cdot V\right)}^{a_{7}},a_{8}\} \end{array}.$$

The finally obtained model coefficients are shown in Table 2.

## 4. Proposed Joint Source and Channel Rate Allocation Scheme

In the MIoT system, multiple mobile camera nodes have to compete for the limited wireless transmission data rate. Due to the packet loss in the wireless network transmission, FEC needs to be used for source data protection. Therefore, we develop a joint source and channel rate allocation scheme for both video and FEC rate allocation with the objective to maximize the product of tracking precision of each uploaded GOP. Let M be the number of mobile camera nodes in system, and **k** and **n** are the M×1 vectors. The element $k_{m}$ in **k** represents the source packets of mobile camera node *m*. The element $n_{m}$ in **n** represents the FEC packets of mobile camera node *m*, which contains the source packets and the protect packets. The proposed scheme allocates the appropriate source and FEC packets for GOP in each camera node, such that the product of the tracking precision of all GOPs is maximized. The joint source and channel rate allocation scheme can be formulated as follows:

(16) $$\begin{array}{l} \underset{n,k}{\max}\;\prod_{m=1}^{M}P_{m}\left(\frac{k_{m}\cdot S}{N_{GOP}\cdot N_{px}}\right)\cdot f(k_{m},n_{m};p_{m})\\ s.t.\;\sum_{m=1}^{M}n_{m}\cdot S\cdot f_{ps}/N_{GOP}\leq R_{total}\\ P_{m}\left(\frac{k_{m}\cdot S}{N_{GOP}\cdot N_{px}}\right)\geq P_{\min}\;\forall m\\ n_{m}\geq k_{m}\;\forall m \end{array},$$

where $p_{m}$ is the packet loss rate of the camera node *m*. *S* is the packet size and $N_{GOP}$ is the GOP size, $R_{total}$ is the total available rate, and $P_{\min}$ is minimum acceptable precision. $P_{m}(\cdot )$ is the tracking precision prediction model established in Section 3, and f(⋅) is the FEC packets correction rate of the camera node *m* in application layer [32]:

(17) $$f(k,n;p)=\Phi \left(\frac{n-k-np}{\sqrt{np(1-p)}}\right)=\frac{1}{\sqrt{2\pi }}\int_{-\infty }^{\frac{n-k-np}{\sqrt{np(1-p)}}}e^{-t^{2}/2}dt,$$

where Φ(⋅) is the cumulative distribution function of the standard normal distribution.

The first constraint indicates that the sum of the uploaded data in a GOP time period is limited by the total data rate. The second constraint implies that the tracking precision of each camera node should meet the predefined minimum requirement. The last constraint means that the number of source packets of camera node m cannot be greater than FEC packets of that camera node. To solve the optimization problem, the logarithm of objective function is employed, and the $k_{m}$ and $n_{m}$ are substituted by ${\tilde{k}}_{m}=\sqrt{k_{m}}$, ${\tilde{n}}_{m}=\sqrt{n_{m}}$. Then the original problem can be formulated as:

(18) $$\begin{array}{l} \underset{n,k}{\max}\;\sum_{m=1}^{M}\left(\log\left(P_{m}\left(\frac{{\tilde{k}}_{m}^{2}\cdot S}{N_{GOP}\cdot N_{px}}\right)\right)+\log\left(f({\tilde{k}}_{m}^{2},{\tilde{n}}_{m}^{2};p_{m})\right)\right)\\ s.t.\;\sum_{m=1}^{M}{\tilde{n}}_{m}^{2}\cdot S\cdot f_{ps}/N_{GOP}\leq R_{total}\\ P_{m}\left(\frac{{\tilde{k}}_{m}^{2}\cdot S}{N_{GOP}\cdot N_{px}}\right)\geq P_{\min}\;\forall m\\ \;{\tilde{n}}_{m}\geq {\tilde{k}}_{m}\;\forall m \end{array}.$$

The reformulated problem is a convex optimization problem and can be solved by convex optimization tools like CVX toolbox [38]. The optimized resolution k***** and n***** are the source packets and the FEC packets for each camera node. Although packets number should be integer in the reality, the elements in k***** and n***** are not required to be integer when solving the problem. The reason for this is that the target source coding rate and FEC coding rate of GOP which are converted from k***** and n***** are allowed to be slightly differ from the actual coding rate.

## 5. Simulation and Performance Comparison

### 5.1. Simulation Settings

**Figure 9:**
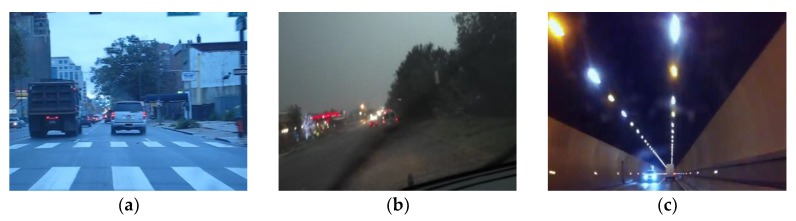

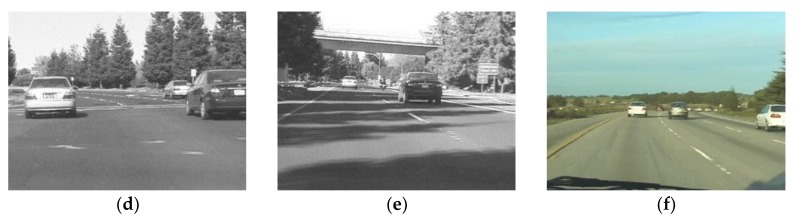
Sample frames of test video sequences: (**a**) BlurCar1(2); (**b**) Wiper; (**c**) Tunnel; (**d**) Car1(1); (**e**) Car1(2); (**f**) Car24.

We simulate a scenario where six mobile camera nodes capture different scenes and upload videos to a server via lossy wireless networks. The six mobile camera nodes have to compete for the limited transmission resource which in our simulation is represented by the total transmission bitrates. The channel condition of each camera node is represented by its packet loss rate. Each camera node extracts the two content features (*L* and *V*) of the current raw GOP and sends these features to the server. The server jointly optimizes the source coding rate and FEC rate for all camera nodes based on these features and packet loss rates of all camera nodes. Then the optimal source coding rates and FEC rates are fed back to each camera node for current GOP coding. The encoded GOPs of six camera node are uploaded to the server. Next GOPs are also processed like this. Finally, we evaluate the tracking performance on the uploaded GOPs. In our simulation, we selected six video sequences from OOTB [37] and visual object tracking (VOT) [39] data set as captured videos of six camera nodes, the video information is shown in Table 3. Here, BlurCar(2) has the 390th to 709th frames in BlurCar1, Car1(1) has the 1st to 320th frames in Car1, and Car1(2) has the 410th to 729th frames in Car1. The sample frames of the six test videos are shown in Figure 9. The FFMPEG implementation is employed as H.264 video encoder. The frame rate and GOP size are 30 fps and 16, respectively. The rate that assigned to a GOP by the different mechanisms is then allocated to frames within the GOP using basic variable bit rate mechanism in the H.264 coder. The GOP structure is IPPP. Each test video has 19 GOPs. The total transmission bit rates change from 400 kb/s to 1400 kb/s, and the change step is 200 kb/s. The content information of each video is shown in Table 3. The packet loss rate is set as 1% or 3% for different videos, and the packet loss rates of GOPs within the video are fixed for comparison purposes.

We compare our proposed scheme with three schemes: the mean square error (MSE) driven rate allocation scheme, QoE driven rate allocation scheme and constant bitrate control (CBR) scheme. In CBR scheme, the total transmission rate is equally allocated to each camera node, and the packet correct rate of each node is set to be 0.995. For the MSE-driven and QoE-driven schemes, we adopt a classical rate-distortion model in [40] and a QoE model in [41]. The corresponding rate allocation problem is expressed as:

(19) $$\begin{array}{l} \underset{n,k}{\min}\;\sum_{m=1}^{M}Q_{m}\left(\frac{k_{m}\cdot S}{N_{GOP}\cdot N_{px}}\right)\\ s.t.\;\sum_{m=1}^{M}n_{m}\cdot S\cdot f_{ps}/N_{GOP}\leq R_{total}\\ \frac{k_{m}\cdot S}{N_{GOP}\cdot N_{px}}\geq R_{\min}\;\forall m\\ \;f(k_{m},n_{m};p_{m})\geq p_{\min}\;\forall m\\ \;n_{m}\geq k_{m}\;\forall m \end{array},$$

here $Q_{m}(\cdot )$ is the MSE/QoE of camera node *m*. $R_{\min}$ is the minimum data rate and is set to be 32 kb/s. $p_{\min}$ is minimum packet correct rate which is set to be 0.995. 

### 5.2. Simulation Results and Discussion

**Figure 10:**
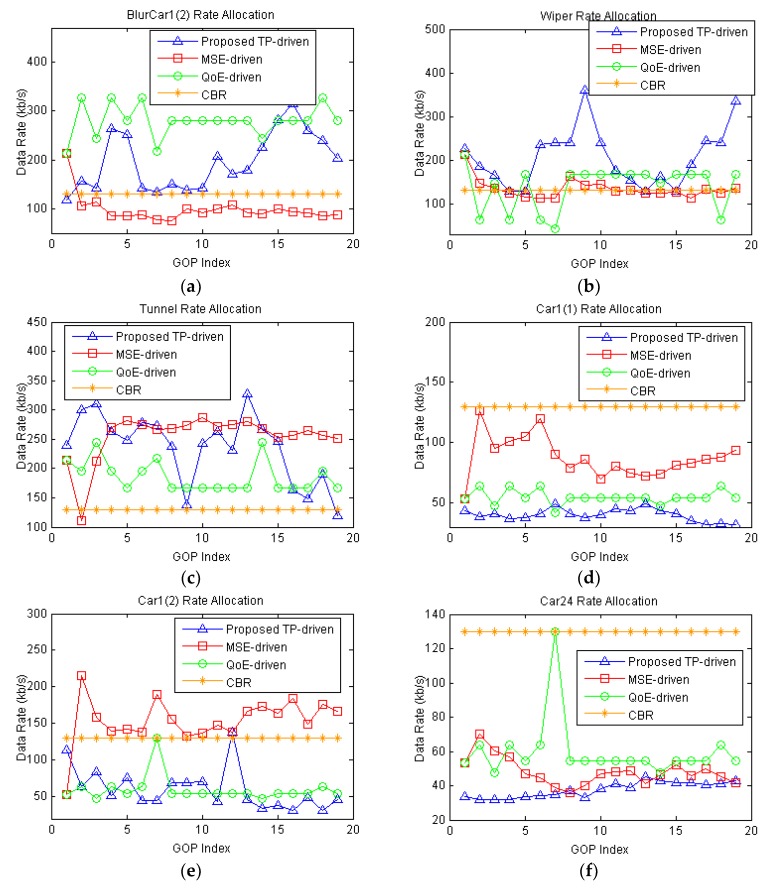
Source coding rate of all GOPs in each video under 800 kb/s total rate constraint. The packet loss rate of each video is 1%: (**a**) BlurCar1(2); (**b**) Wiper; (**c**) Tunnel; (**d**) Car1(1); (**e**) Car1(2); (**f**) Car24.

We set a fixed total data rate of 800 kb/s for the proposed tracking-precision-driven scheme (TP-driven scheme), MSE-driven scheme, QoE-driven scheme and CBR scheme. The packet loss rates of all camera nodes are set to be 1%. Figure 10 shows the source data rate of each GOP. The proposed TP-driven scheme allocates less data rate to the GOPs which have high luminance level and adjacent block difference (like GOPs in Car1(2) and Car24) to save bit rates. For the GOPs with low luminance level or adjacent block difference, the proposed TP-driven scheme allocates more data rates to these GOPs to increase video quality for vehicle tracking purpose (5th to 12th GOPs in Wiper, 15th to 18th in BlurCar1(2)). The MSE-driven scheme allocates more data rates to the GOPs with more objects or details. For example, the Car1(1) and Car1(2) are allocated more rates than Car24 by MSE-driven scheme. However, the MSE-driven cannot distinguish the GOPs by video content, and therefore GOPs in BlurCar1(2) and Car1(2) are allocated similar date rate even these GOPs have different degrees of tracking difficulty. The QoE model classifies GOPs into three different content type, ’slight movement’, ‘gentle walking’ and ‘rapid movement’, then the QoE-driven scheme allocates more data rates to GOPs with ‘rapid movement’ content type and less data rates to GOPs with ‘slight movement’ content type. Therefore, compared with MSE-driven and TP-driven scheme, QoE-driven scheme allocates more data rates to BlurCar1(2) because it is ‘rapid movement’.

**Figure 11:**
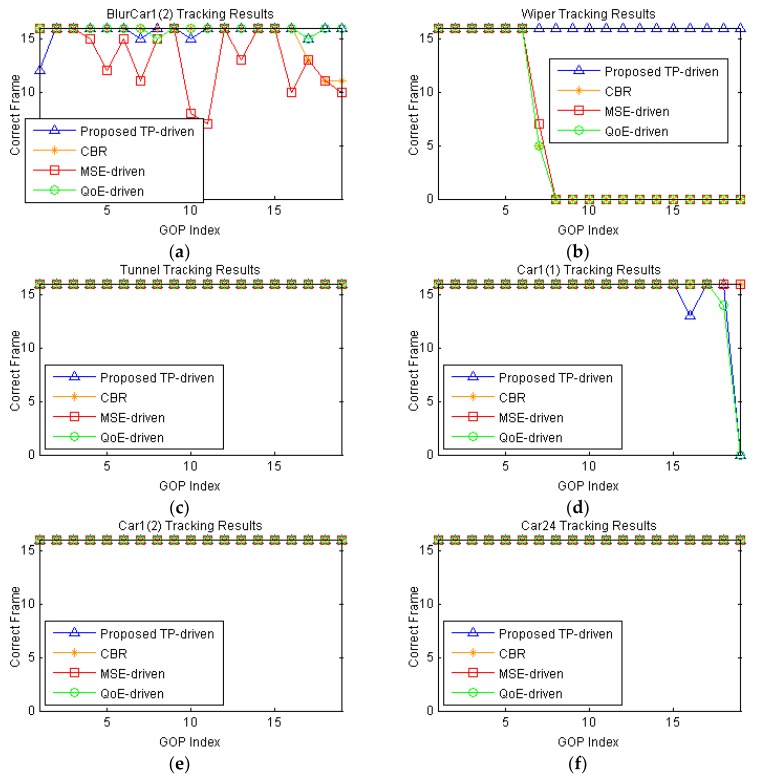
Tracking results of all GOPs in each video under 800 kb/s total rate constraint. The packet loss rate of each video is 1%: (**a**) BlurCar1(2); (**b**) Wiper; (**c**) Tunnel; (**d**) Car1(1); (**e**) Car1(2); (**f**) Car24.

Next, we conduct tracking on these video sequences by KCF scheme. The number of correct tracking frames in each GOP is illustrated in Figure 11. 

Here, we assume that the ground truth of the first frame in each video sequence is assigned by the server. From Figure 11, we can see that our proposed TP-driven scheme allocates the bit rates more efficiently. For instance, in the Wiper video sequence, the KCF tracks the right target in GOPs from the proposed scheme while it loses the target in GOPs encoded by the MSE-driven, QoE-driven and CBR schemes. In the BlurCar1(2) video, the proposed scheme achieves higher tracking precision than the MSE-driven and CBR schemes. This is because the proposed scheme considers content the impacts on tracking and allocates more data rates to these GOPs to provide high tracking precision. For Car24, all schemes get the same tracking precision, but the proposed TP-driven scheme saves more bit rates. Although the proposed scheme allocates less rates to the Car1(1) and sacrifices tracking performance in its GOPs, the overall performance in the six test video is better than the MSE-driven scheme, QoE-driven scheme and CBR scheme.

**Figure 12:**
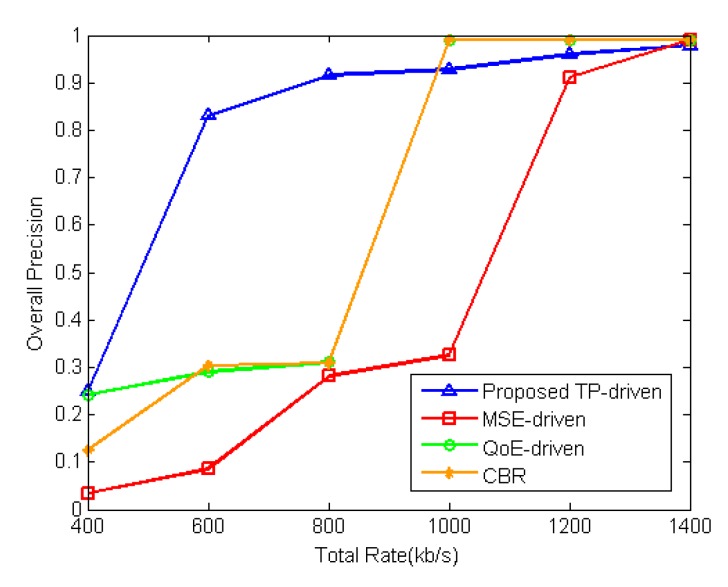
Product of tracking precision of each video under different total rate constraints. The packet loss rate of each video is 1%.

**Figure 13:**
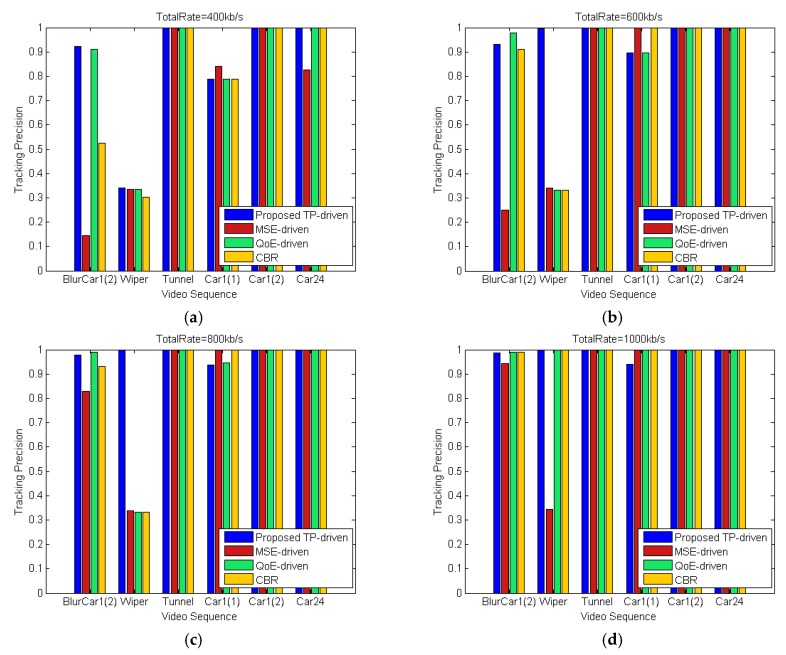

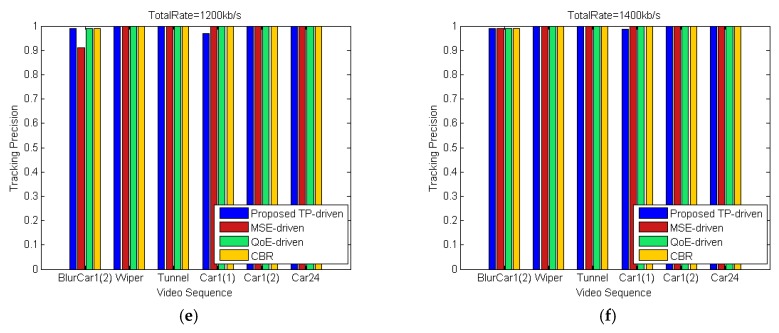
Tracking precision of each video under different total rate constraints. The packet loss of each video is 1%: (**a**) Total rate = 400 kb/s; (**b**) Total rate = 600 kb/s; (**c**) Total rate = 800 kb/s; (**d**) Total rate = 1000 kb/s; (**e**) Total rate = 1200 kb/s; (**f**) Total rate = 1400 kb/s.

The performance of the proposed TP-driven scheme, MSE-driven scheme, QoE-driven scheme and CBR scheme is tested under different total rate constraints. Figure 12 shows the product of tracking precisions of all video sequences under different total rate constraints. Figure 13 illustrates the detailed tracking precision of each video under different total rate constraints. The packet loss rates are set to be 1% for all mobile cameras. The proposed TP-driven scheme gets better performance when the total rate is limited and deficient. For example, as shown in Figure 13, when the total rate is 800 kb/s, the proposed scheme achieves higher tracking precision for BlurCar1(2) and Wiper. For Tunnel, Car1(2) and Car24, the four schemes show little tracking precision differences. For Car1(1), the proposed scheme gets lower tracking precision than the other schemes. However, the products of tracking precision of all video sequences under the proposed TP-drive, MSE-driven, QoE-driven and CBR scheme are 0.92, 0.28 0.29 and 0.31, respectively. It is obvious that the tracking precision of the MSE-driven, QoE-driven and CBR schemes is lower than that of the proposed scheme as shown in Figure 12. Therefore, the proposed TP-driven scheme achieves better overall performance in vehicle tracking.

**Figure 14:**
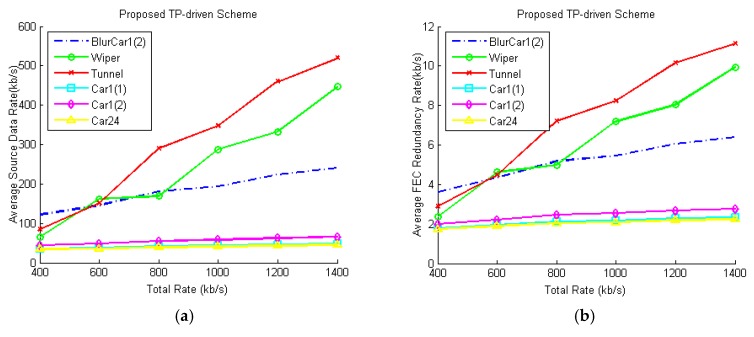

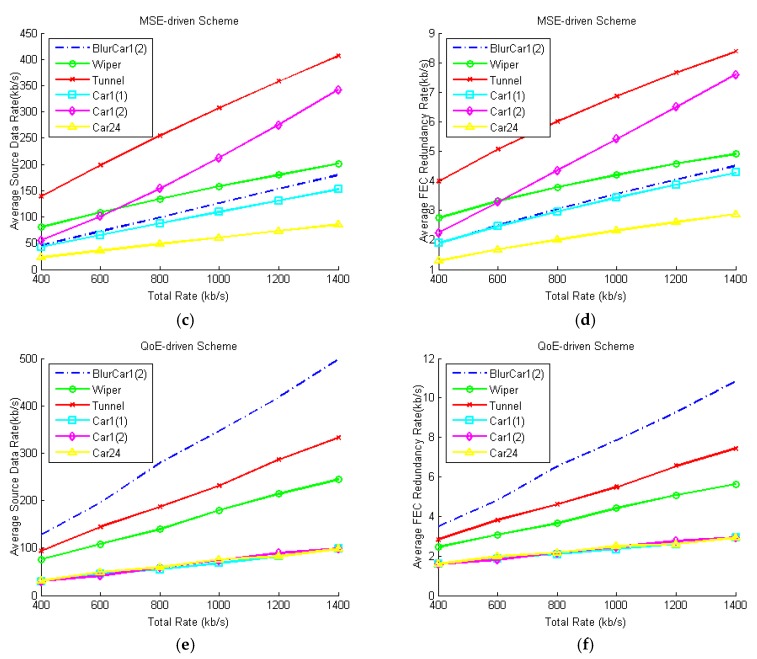
Average source data rates and FEC redundancy rates of each video under different total rate constraints. The packet loss rate of each video is 1%: (**a**) Average source data rates of proposed scheme; (**b**) Average FEC redundancy rates of proposed scheme; (**c**) Average source data rates of MSE-driven scheme; (**d**) Average FEC redundancy rates of MSE-driven scheme; (**e**) Average source data rates of QoE-driven scheme; (**f**) Average FEC redundancy rates of QoE-driven scheme.

**Figure 15:**
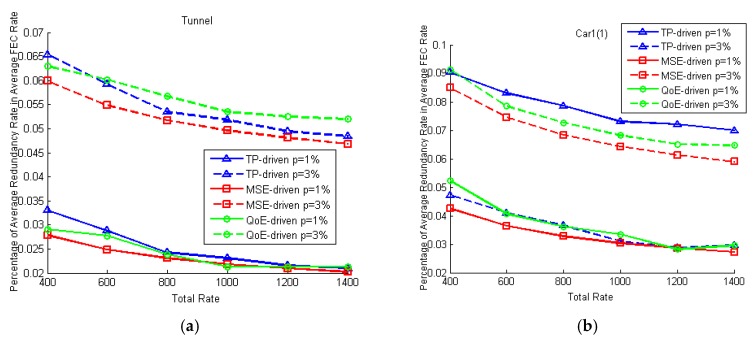
Percentage of FEC redundancy rates in total coding rates of Tunnel and Car1(1) under different rate constraints. In the fisrt case (solid lines), packet loss rate of each videos is 1%. In the second case (broken lines), packet loss rates of Tunnel and Car1(1) are 3%, and packet loss rates of other videos are 1%: (**a**) Percentage of FEC redundancy rates in total coding rates of Tunnel; (**b**) Percentage of FEC redundancy rates in total coding rates of Car1(1).

**Figure 16:**
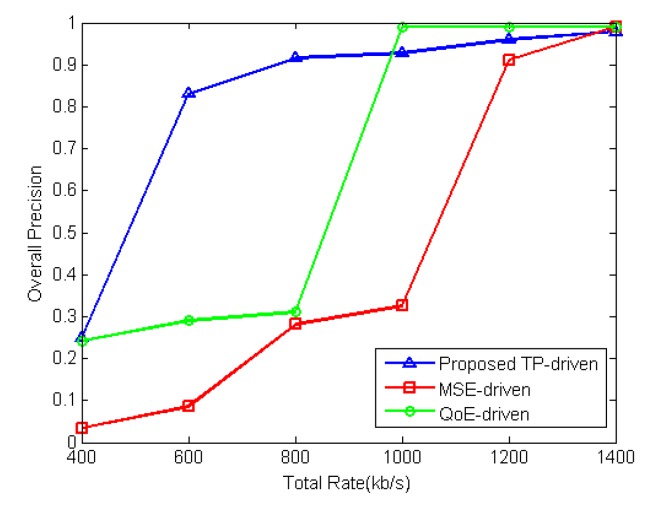
Product of tracking precision of each video under different total rate constraints. The packet loss rates of Tunnel and Car1(1) are 3%, and packet loss rates of other videos are 1%.

We test the FEC rate allocation by setting different packet loss rates for each video. Since in the CBR scheme, the source data rates of all camera nodes are equally allocated to all videos, only the TP-driven, MSE-driven and QoE-driven schemes are tested. In the first case, the packet loss of each video is 1%. Figure 14 shows the average source data rate and average FEC redundancy rate under different total rate constraints in this case. We can see that videos are allocated more redundancy rates with the increase of the total rate. The proposed TP-driven scheme allocates more redundancy rates to the videos with low luminance level and adjacent block differences to avoid packet losses. Meanwhile the MSE-driven and QoE-driven schemes are prone to protect the video with more details and rapid movement, respectively. Then the packet loss rates of Tunnel and Car1(1) are set as 3%, the packet loss rates of other videos are set as 1%. Figure 15 shows the percentage of redundancy rates in the total coding rates of Tunnel and Car1(1) in the two cases. As shown in Figure 15, all schemes allocate more data to videos with 3% packet loss rates to protect the data packets. From Figure 15 we can see that although the redundancy rates increase when the total constraint rates increase as shown in Figure 14, the percentage of redundancy rates decrease with the increase of total bit rates. This is because the increase of the redundancy rates is lower than the increase of the source data rates. The product of tracking precision of each video in this case is shown in Figure 16, which is almost the same as the product in the first case. It is because the changes of source data rates and FEC packets correction rates that caused by packet loss rate changes are very slight.

In our simulation, the GOP structure is IPPP. In fact, several B frames are allowed in the GOP, GOP structure like IBBPBBP can provide similar performance like IPPP. However, the GOP structures which have a lot of consecutive B frames (IBB…BP structure for example) are not suitable for our scheme. Due to the blurring and fast movement in the video, a lot of consecutive B frames can cause obvious video quality degradation. Consequently, tracking precision decreases. Therefore, I or P frames are needed to be inserted every several B frames in the GOP for high quality service.

## 6. Conclusions

In this paper, we have proposed a joint source and channel coding algorithm for vehicle tracking in MIoT systems. Our algorithm aims to provide better tracking performance in MIoT servers, which is different with previous QoS/QoE-driven schemes which aim to improve the human visual experience. We first study the effect of video content and compression on the KCF tracking scheme and establish a content-aware tracking precision prediction model, which can estimate the tracking precision of videos with different content features and coding rates. In this model, video luminance level and adjacent block difference are extracted to represent the effect of lighting conditions and video content complexity on tracking precision, respectively. Then, based on the precision prediction model, the optimization problem is formulated to maximize the product of tracking precision of each surveillance video under the constraint of the total transmission rate in the wireless network. Considered the transmission error in wireless network, FEC is adopted to improve the reliability of data transmission. Compared with the traditional QoS/QoE-driven algorithm and CBR algorithm, our joint source and channel rate allocation algorithm more efficiently allocates the bit rates under the constraint total transmission rate and achieve better tracking performance.

## Figures and Tables

**Table 1:** The encoded bitrate of videos.

Video	Resolution	Bitrate(kb/s)					
BlurCar1(1)	640 × 480	32	64	128	256	512	768
BlurCar3	640 × 480	32	64	128	256	512	768
BlurCar4	640 × 480	32	64	128	256	512	768
Car2	320 × 240	16	32	64	128	256	512
Car4	360 × 240	16	32	64	128	256	512
CarDark	320 × 240	16	32	64	128	256	512

**Table 2:** Model coefficients.

***a*_1_**	***a*_2_**	***a*_3_**	***a*_4_**
$1.049\times 10^{-15}$	12.661	7.034	200.560
***a*_5_**	***a*_6_**	***a*_7_**	***a*_8_**
0.010	0.900	6.150	137.000

**Table 3:** Test video sequences.

Video	Resolution	Lighting Condition	Content Complexity
BlurCar1(2)	640 × 480	Medium	Medium
Wiper	640 × 480	Low	High
Tunnel	640 × 360	Low	Medium
Car1(1)	320 × 240	High	Medium
Car1(2)	320 × 240	High	Low
Car24	320 × 240	High	Low
